# Cognitive and Affective Empathy Relate Differentially to Emotion Regulation

**DOI:** 10.1007/s42761-021-00062-w

**Published:** 2021-11-15

**Authors:** Nicholas M. Thompson, Carien M. van Reekum, Bhismadev Chakrabarti

**Affiliations:** School of Psychology and Clinical Language Sciences, University of Reading, Reading, RG6 6AL UK

**Keywords:** Empathy, Emotion regulation, Cognitive, Affective, Social cognition

## Abstract

The constructs of empathy (i.e., understanding and/or sharing another’s emotion) and emotion regulation (i.e., the processes by which one manages emotions) have largely been studied in relative isolation of one another. To better understand the interrelationships between their various component processes, this manuscript reports two studies that examined the relationship between empathy and emotion regulation using a combination of self-report and task measures. In study 1 (*N* = 137), trait cognitive empathy and affective empathy were found to share divergent relationships with self-reported emotion dysregulation. Trait emotion dysregulation was negatively related to cognitive empathy but did not show a significant relationship with affective empathy. In the second study (*N* = 92), the magnitude of emotion interference effects (i.e., the extent to which inhibitory control was impacted by emotional relative to neutral stimuli) in variants of a Go/NoGo and Stroop task were used as proxy measures of implicit emotion regulation abilities. Trait cognitive and affective empathy were differentially related to both task metrics. Higher affective empathy was associated with increased emotional interference in the Emotional Go/NoGo task; no such relationship was observed for trait cognitive empathy. In the Emotional Stroop task, higher cognitive empathy was associated with reduced emotional interference; no such relationship was observed for affective empathy. Together, these studies demonstrate that greater cognitive empathy was broadly associated with improved emotion regulation abilities, while greater affective empathy was typically associated with increased difficulties with emotion regulation. These findings point to the need for assessing the different components of empathy in psychopathological conditions marked by difficulties in emotion regulation.

**Supplementary Information:**

The online version contains supplementary material available at 10.1007/s42761-021-00062-w.

Our social lives often rely upon the linked processes of how we manage our own emotions and respond to the emotions of others. Yet, until recently the constructs of empathy (i.e., understanding and/or sharing another’s emotion) and emotion regulation (i.e., the processes by which one manages emotions) have largely been studied in relative isolation of one another. Despite the growing research interest in this area, fundamental questions remain regarding the nature of the interrelationships between the various component processes that comprise empathy and emotion regulation. To address these gaps in current knowledge, this manuscript reports two studies which examined how trait empathy is associated with self-report (study 1) and task-based (study 2) measures of emotion regulation.

Empathy is commonly conceptualised as comprising two dimensions: affective empathy (the ability to *share* others’ emotions), and cognitive empathy (the ability to infer/*understand* others’ emotional experiences; Chakrabarti & Baron-Cohen, 2006; Decety & Jackson, 2004; Singer & Lamm, 2009). Emotion regulation refers to the various processes that can be employed in order to influence one’s own emotions (Gross, 2015). A close relationship between empathy and emotion regulation has been proposed by previous theoretical accounts, including our own (e.g., Decety, 2010; Schipper & Peterman, 2013; Thompson et al., 2019; Zaki, 2014). Of the handful of empirical studies that have explored this relationship, most have focused on the moderating role of emotion regulation on the association between affective empathy and different ‘empathic outcomes’, such as empathic concern (i.e., sympathy), personal distress, and prosocial behaviours (e.g., Brethel-Haurwitz et al., 2020; Lockwood et al., 2014; Lopez-Perez & Ambrona, 2015; Okun et al., 2000). While not their primary focus, such studies provide evidence suggestive of a direct relationship between empathy and emotion regulation. For example, one’s self-reported capacity to understand others’ emotions is positively related to the habitual use of more adaptive emotion regulation strategies such as reappraisal (Lockwood et al., 2014; Powell, 2018; Tully et al., 2016). Similarly, self-reported perspective-taking ability (a process associated with cognitive empathy) is negatively related to trait measures of emotion dysregulation (Contardi et al., 2016; Eisenberg & Okun, 1996; Okun et al., 2000).

Some of this prior work suggests a potential overlap in the cognitive control processes that underlie key abilities associated with these constructs. The ability to attribute an emotional/mental state to another individual (i.e., cognitive empathy) requires various cognitive control processes, which facilitate the necessary coordination of self and other representations (e.g., Carlson et al., 2004; Decety & Sommerville, 2003; Hansen, 2011; Mutter et al., 2006). Similar cognitive control processes have been implicated in emotion regulation, where they underlie the ability to exert control over one’s affective responses and behaviours (e.g., Buhle et al., 2014; Hendricks & Buchanan, 2016; McRae et al., 2012; Schmeichel & Demaree, 2010).

While the aforementioned evidence would suggest that greater cognitive empathy may facilitate emotion regulation, there is reason to expect that increased affective empathy could in fact hinder these processes. Individuals with higher affective empathy exhibit an increased facial mimicry response to others’ emotions (Sonnby-Borgstrom, 2002; Dimberg et al., 2011), which has been shown to relate to heightened resonance with the mimicked emotion (Hatfield et al., 1993; Laird et al., 1994; Wild et al., 2001). Additionally, individuals with greater affective empathy may be predisposed to experience emotions with a greater intensity and/or frequency (Davis, 1983; Eisenberg et al., 2000; Rueckert et al., 2011; Sato et al., 2013). Given that emotions can have a detrimental effect on the efficiency of cognitive control processes (Hare et al., 2008; Tottenham et al., 2011), it is possible that the increased emotional reactivity associated with higher levels of affective empathy could interfere with one’s ability to engage the often demanding processes necessary for emotion regulation*.*

Building upon prior work, the current research adopted a multi-method approach in which we examined the relationship between empathy and emotion regulation using a combination of self-report and more objective performance-based measures. Study 1 examined how trait cognitive empathy and affective empathy are associated with self-reported difficulties with emotion regulation (i.e., emotion dysregulation). Study 2 examined how the same trait empathy measure relates to task metrics that index emotion regulation abilities. Based on the findings discussed above, it was predicted that higher cognitive empathy would be associated with improved emotion regulation, whereas the opposite relationship would be observed for affective empathy.

## Study 1

Various abilities should be taken into account when considering an individual’s capacity for emotion regulation, including (1) awareness/understanding of one’s emotions, (2) the capacity to select and implement appropriate regulation strategies to manage emotions across diverse contexts, and (3) the ability to monitor the extent to which regulatory efforts successfully generate the desired modulation of emotion (Bonanno & Burton, 2013; Gohm & Clore, 2002; Gratz & Roemer, 2004; Gross, 2015; Koole et al., 2015). In this first study, we examined how trait cognitive empathy and affective empathy are related to a self-report measure of emotion regulation that assesses difficulties with emotion regulation across each of the abilities highlighted above (Kaufman et al., 2016). We predicted that self-reported emotion dysregulation would be negatively related to trait cognitive empathy but show a positive relationship with trait affective empathy.

## Methods

### Participants

An a priori sample size estimation was conducted using G*power 3.1 (Faul et al., 2007). Based on an expected correlation coefficient of ~ 0.3 (Contardi et al., 2016; Lockwood et al., 2014), a minimum sample of 67 was required to detect such effects at an alpha level of *p* = 0.05, with a power of 0.80. To account for the potential of data loss due to incomplete responses in the online survey, we aimed to collect a sample of approximately *N* = 100. A sample of 137 participants (101 female), with a mean age (± SD) of 20.6 years (± 3.01), was recruited from the University of Reading campus via the school of psychology research panel. Questionnaires were completed online and participants were awarded course credit for participation. Ethical approval was obtained from the University of Reading research ethics committee. This study was conducted as part of a larger project and included an additional questionnaire measure of emotion regulation (the ERQ; Gross & John, 2003), the data from which are not reported here.

### Materials

#### Empathy

Trait empathy was measured using the Questionnaire of Cognitive and Affective Empathy (QCAE; Reniers et al., 2011). The QCAE is a 31-item self-report questionnaire assessing respondents’ capacity to understand and resonate with the emotions of others. It comprises five subscales which track onto the two dimensions of empathy (cognitive and affective). The cognitive empathy dimension assesses one’s propensity to take another’s perspective and accurately infer their state (e.g., “When I am upset at someone, I usually try to ‘put myself in his shoes’ for a while”; “I can sense if I am intruding, even if the other person does not tell me”). The affective empathy dimension comprises items assessing respondents’ tendency to resonate with others’ emotions (e.g., “I am happy when I am with a cheerful group and sad when the others are glum”; “It affects me very much when one of my friends seems upset”). Participants rate their response to each item using a 4-point scale, ranging from “strongly disagree” to “strongly agree”; higher values reflect greater trait empathy. Cronbach’s alpha was high for both empathy dimensions (α_Cognitive empathy_ = 0.88; α_Affective empathy_ = 0.82). The cognitive and affective subscales of the QCAE were positively correlated, *rho*(135) = 0.42 (CI_95%_[0.27, 0.55]), *p* < 0.001.

#### Emotion Dysregulation

Trait emotion dysregulation was measured using the Difficulties in Emotion Regulation-short form (DERS-SF; Kaufman et al., 2016). The DERS-SF (henceforth, DERS) is an 18-item questionnaire assessing difficulties in six aspects of emotion regulation: (1) awareness of emotions (awareness), (2) clarity/understanding of emotions (clarity), (3) acceptance of emotions (non-acceptance), (4) capacity to maintain goal-directed behaviours in emotional situations (goals), (5) ability to exert control over one’s emotional impulses (impulse), and (6) ability to effectively manage one’s emotional responses (strategies). Respondents report the frequency with which they experience difficulties in these aspects of emotion regulation using a 5-point Likert scale, where 1 = almost never (0–10% of the time) and 5 = almost always (91–100% of the time); higher ratings reflect increased emotion dysregulation. The sum of the six subscales provides a total score reflecting overall levels of emotion dysregulation (DERS-Total). Within this sample, the DERS-Total metric and each subscale thereof demonstrated acceptable to high Cronbach’s alpha: α_DERS-Total_ = 0.91; α_Awareness_ = 0.79; α_Clarity_ = 0.86; α_Acceptance_ = 0.82; α_Goals_ = 0.89; α_Impulse_ = 0.89; α_Strategies_ = 0.83.

### Data Reduction and Analysis

The relationship between empathy and emotion dysregulation was examined using bivariate correlations. Normality of each variable was assessed using Kolmogorov–Smirnov tests. As some variable distributions showed significant deviation from normality, Spearman’s rho is reported for all correlations in order to enable their direct comparability. All correlations are reported as two-tailed, with an alpha level of *p* = 0.05. Data were analysed using SPSS version 27 (IBM Corp., Armonk, N.Y., USA) and Jamovi version 1.6 (https://www.jamovi.org). To ensure that the observed results were not unduly influenced by a small number of outlier cases, the results following the removal of univariate and bivariate outliers are reported in supplementary material (S1).

## Results

**Fig. 1:**
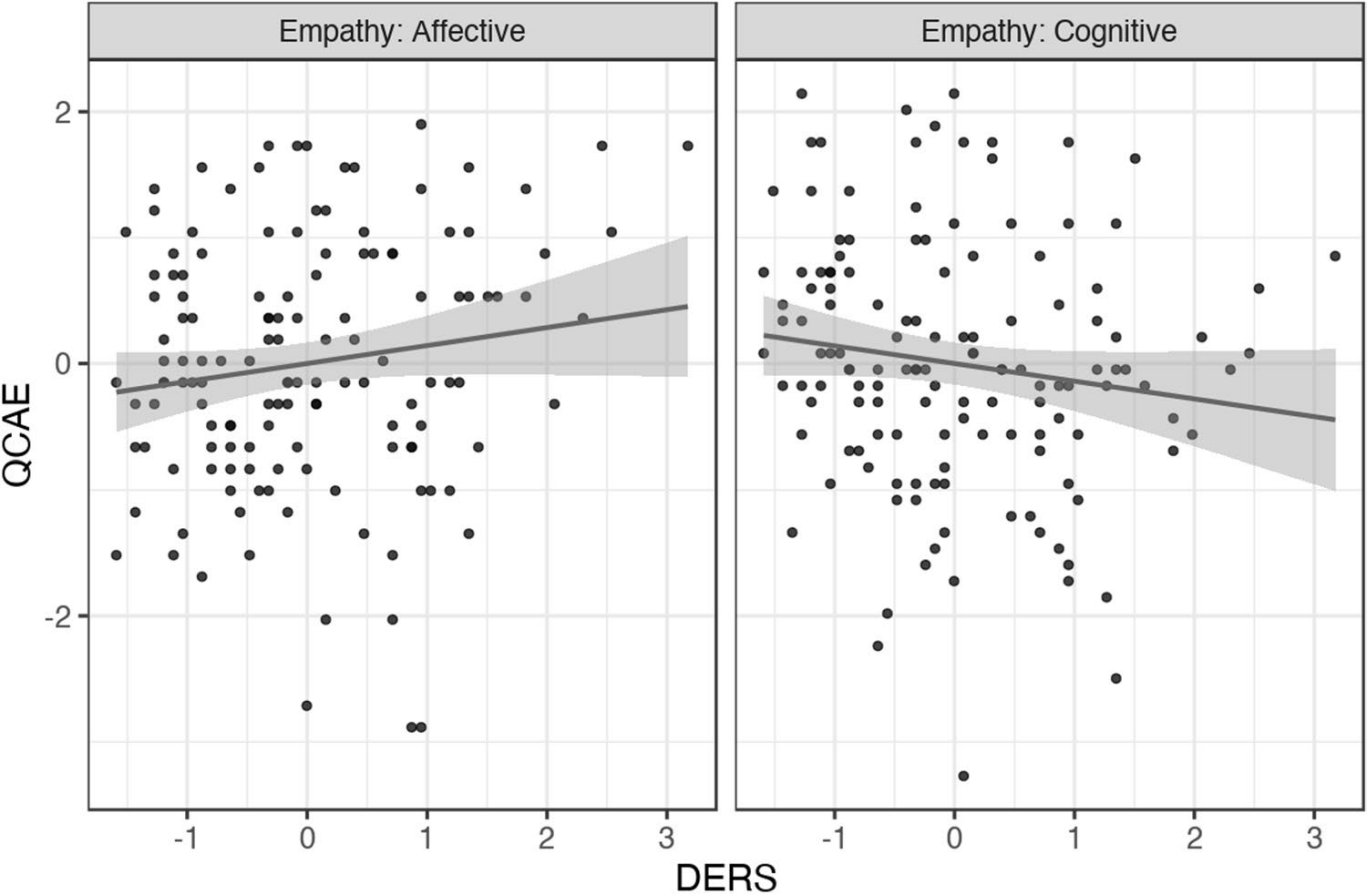
Scatterplot showing the relationship between *Z*-transformed trait cognitive/affective empathy (QCAE) and *Z*-transformed emotion dysregulation (DERS-Total). Affective empathy (left panel) was not significantly correlated with DERS-Total; cognitive empathy (right panel) was negatively correlated with DERS-Total

Descriptive statistics for all variables used in the correlation analysis are reported in Table 1 (descriptive statistics for the DERS subscales are reported in supplementary material, S2). Affective empathy was not significantly correlated with DERS-Total, *rho*(135) = 0.13 (CI_95%_ [− 0.04, 0.29]), *p* = 0.14. Cognitive empathy showed a small significant negative correlation with DERS-Total, *rho*(135) =  − 0.18 (CI_95%_ [− 0.34, − 0.01]), *p* = 0.04. These two correlations were significantly different from each other, Steiger’s *Z* =  − 3.36, *p* < 0.001 (Fig. 1).

**Table 1:** Descriptive statistics for the QCAE subscales and DERS-Total score

	Mean (SD)	Skewness^a^	Kurtosis^b^
QCAE-Cognitive	58.37 (7.76)	− 0.17	0.32
QCAE-Affective	34.88 (5.85)	− 0.34	0.07
DERS-Total	43.04 (12.59)	0.62	− 0.05

^a^Skewness standard error = 0.21.

^b^Kurtosis standard error = 0.41.

To better understand the relationship between cognitive/affective empathy and trait emotion dysregulation, we conducted exploratory correlations testing the relationship between both empathy dimensions and each subscale of the DERS. Cognitive empathy was significantly negatively related to the awareness subscale and showed a trend-level negative relationship with the impulse control subscale. Affective empathy was significantly positively related to the goals and strategies subscales and showed a trend-level positive relationship with non-acceptance. There was also a significant negative relationship between affective empathy and the awareness subscale. Steiger’s tests demonstrated that, with the exception of the clarity subscale, the cognitive and affective dimensions of empathy showed significantly different relationships with each subscale of the DERS. False Discovery Rate (FDR)–corrected correlation results and associated *Z* statistics are reported in Table 2.

**Table 2:** Correlation coefficients for cognitive/affective empathy and DERS subscales

	Awareness	Clarity	Non-acceptance	Goals	Impulse	Strategies
CE^a^	− 0.40***	− 0.16	− 0.15	0.11	* − *0.17	* − *0.10
AE^b^	− 0.22*	0.01	0.16	0.29**	0.04	0.23*
Z (CE:AE)	− 2.07*	*− 1.84*	− 3.36***	− 1.99*	− 2.27*	− 3.59***

^a^Cognitive empathy, ^b^affective empathy.

* < 0.05, ** < 0.01, *** < 0.001.

## Study 2

The results of study 1 highlight several points of divergence between trait cognitive empathy and affective empathy in terms of their relationship with self-reported emotion dysregulation. Cognitive empathy was more negatively related to difficulties with emotional awareness than affective empathy. Significant differences between these empathy components were also observed in relation to other subscales of DERS measuring difficulties with impulse control (impulse), maintaining focus on goal-directed behaviours (goals), and managing/accepting one’s emotions (strategies and non-acceptance, respectively). Many of these abilities are thought to be dependent upon more ‘implicit’ forms of emotion regulation, which refer to regulatory processes that can be enacted with little or no reliance upon conscious effortful control or monitoring (Gyurak et al., 2011; Mauss et al., 2007). Consequently, in this second study, we examined how these same measures of trait cognitive and affective empathy are associated with performance on two tasks that assess implicit emotion regulation abilities.

Prior work has highlighted the deleterious impact of emotional stimuli on cognitive control processes (e.g., Jasinska et al., 2012; Padmala et al., 2011; Reeck & Egner, 2011; Tottenham et al., 2011). While definitions of emotion regulation often emphasise more deliberate and effortful processes, regulating the early influence of emotions on cognitive control is largely dependent upon more implicit processes, which also play a key role in emotion regulation in daily life (Gyurak et al., 2011; Mauss et al., 2007).

Emotional variants of well-known cognitive control tasks are commonly used to assess attentional biases and the interaction between emotion and cognition. Such tasks have also proven useful for assessing more implicit emotion regulation abilities (see review by Buhle et al., 2010). In these paradigms, performance is compared across conditions in which distracting stimuli are either emotional or non-emotional, thereby indexing the extent to which performance is disrupted by emotional information (a phenomenon henceforth referred to as ‘emotion interference effects’). The assumption underlying this approach is that individuals with better implicit emotion regulation abilities will exhibit a reduced magnitude of emotion interference effects relative to their less well-regulated counterparts (Etkin et al., 2010; Koole & Rothermund, 2011; Zhang & Lu, 2012). In the present study, we used two paradigms that have been used in previous research to assess implicit emotion regulation: the emotional go/nogo and the emotional Stroop (henceforth Emo-GNG and Emo-Stroop, respectively). While the term implicit is sometimes used in reference to regulation paradigms wherein the processing of emotional information is entirely irrelevant to task performance (Zhang & Lu, 2012), we use the term slightly more broadly to refer to tasks in which regulatory processes may be initiated without any explicit instruction/intention (Yiend et al., 2008).

Prior studies using the Emo-GNG have demonstrated that emotional nogo stimuli tend to induce more commission errors (i.e., false alarms) relative to neutral nogo stimuli (Hare et al., 2005, 2008; Tottenham et al., 2011; Zhang et al., 2016). Similarly, numerous emotional variants of the Stroop task (Stroop, 1935) have demonstrated the heightened potential for emotional stimuli to disrupt cognitive control processes relative to neutral stimuli, indexed by increased response times (RT) and/or decreased accuracy (Etkin et al., 2006; Haas et al., 2006; Stenberg et al., 1998). These emotion interference effects are often exhibited to a greater extent, or in some cases, only, in individuals with high trait anxiety (e.g., Kalanthroff et al., 2016; Richards et al., 1992; see also Buhle et al., 2010).

One prior study found that improved performance on a task measuring cognitive empathy–related processes was associated with a greater ability to ignore irrelevant distractors in an Emo-Stroop (Bradford, 2015). However, this study did not test the relationship between affective empathy and the Emo-Stroop. Additionally, while there is some evidence to suggest that affective empathy is associated with a heightened sensitivity to emotional stimuli (such as in attentional blink tasks, e.g., Kanske et al., 2013), prior work has failed to find any direct evidence for a relationship between affective empathy and interference effects in Emo-Stroop tasks (Hofelich & Preston, 2012).

It is important to note that the trait empathy measures used in these prior studies have been criticised for conflating the cognitive and affective dimensions of empathy with dissociable constructs, such as sympathy (Reniers et al., 2011). Furthermore, many studies that have used Emo-Stroop variants lacked a neutral control condition, which makes it difficult to determine the extent to which any RT difference between the congruent and incongruent conditions is driven by emotion interference effects (on incongruent trials) or emotion facilitation effects (on congruent trials). Accordingly, the current study includes a neutral control condition to enable the examination of emotional interference independently of facilitation. As prior work has demonstrated the potential for both positively and negatively valenced stimuli to attract attention and disrupt cognitive processing (e.g., Hare et al., 2005; Pratto & John, 1991), both tasks included positive and negative emotional facial expressions in order to increase the generalizability of the results.

It was expected that emotional stimuli would disrupt inhibitory control processes, as indexed by decreased performance relative to neutral stimuli (i.e., increased emotion interference effects). Based on the literature discussed in the general introduction, it was predicted that trait cognitive and affective empathy would be differentially related to the magnitude of these emotion interference effects. Given evidence suggestive of overlap in the cognitive control processes underlying abilities related to cognitive empathy and emotion regulation (e.g., Buhle et al., 2014; Decety & Sommerville, 2003; Hansen, 2011; Hendricks & Buchanan, 2016), it was predicted that cognitive empathy would be negatively related to the magnitude of emotion interference effects. Conversely, based on evidence that affective empathy is positively associated with increased spontaneous mimicry and emotional reactivity (Davis, 1983; Eisenberg et al., 2000; Hatfield et al., 1993; Laird et al., 1994; Wild et al., 2001), it was predicted that trait affective empathy would show a positive relationship with the magnitude of emotion interference effects.

## Methods

### Participants

As in study 1, an a priori sample size estimation was conducted using G*power 3.1 (Faul et al., 2007). Based on an expected correlation coefficient of ~ 0.3 (Contardi et al., 2016; Lockwood et al., 2014), a minimum sample of 67 was required to detect such effects at an alpha level of *p* = 0.05, with a power of 0.80. To account for potential data loss due to non-responders and data outliers, we sought to obtain a sample of approximately *N* = 100. Ninety-two right-handed participants (78 females) were recruited from the undergraduate psychology population at the University of Reading. All participants were recruited through the online research panel and received course credit for participation. The mean age (± SD) was 19.86 (± 2.39). The two tasks were administered as part of a lab session, with the order of task completion counterbalanced. This study was conducted as part of a larger project and included an additional questionnaire measure of emotion regulation (the ERQ; Gross & John, 2003), the data from which are not reported here.

Following data quality checks (see “Data Reduction and Analysis” for details), thirteen participants’ data were removed from the Emo-GNG, and nine participants’ data were removed from the Emo-Stroop, leaving a final sample of *N* = 79 (71 females; mean age ± SD = 19.95 ± 2.55) and *N* = 83 (69 female; mean age ± SD = 19.93 ± 2.50) for these two tasks, respectively. The QCAE was completed online; Cronbach’s alpha was high for both QCAE subscales (α_Affective Empathy_ = 0.79; α_Cognitive Empathy_ = 0.88). Following case removals based on the Emo-GNG and Emo-Stroop task data, the cognitive and affective subscales of the QCAE were positively correlated in both instances, *rho*(77) = 0.38 (CI_95%_[0.18, 0.56]), *p* < 0.001; *rho*(81) = 0.36 (CI_95%_[0.15, 0.53]), *p* = 0.001.

### Materials and Procedure

#### Emo-GNG

Face stimuli were taken from the Nimstim Face Stimulus Set (Tottenham et al., 2009; www.macbrain.org) and comprised photographs of six female (identity numbers: 1, 3, 6, 8, 9, 10) and six male (identity numbers: 21, 22, 23, 24, 28, 34) actors. Different male and female actors displaying fearful expressions were used for the practice block. Each image was converted to greyscale with the dimensions 256 × 329 pixels. The facial expressions included in the task were the closed-mouth versions of happy, sad, disgusted, and calm (i.e., neutral). These emotions were selected in order to include stimuli depicting expressions of positive, neutral, and negative valence, which were likely to differ in terms of the approach/avoid tendencies they elicit (Seidel et al., 2010; Tottenham et al., 2011). The same actors were used for each facial expression, and the frequency with which each stimulus was presented was balanced across conditions.

The Emo-GNG task lasted approximately 25 min. This included a practice block comprising 12 trials, followed by 6 experimental blocks each comprising 48 trials. Upon completion of a block, a holding screen was presented until participants pressed a key to continue; the order in which the blocks were completed was randomised. In each block, an emotional target (go) or distractor (nogo) was always paired with a calm target/distractor, such that if an emotional face was the go stimulus, a calm face was the nogo stimulus, and vice versa. Each block contained one emotion type only, alongside the neutral (calm) expressions. At the start of each block, participants were told which emotion represented the go stimulus and were instructed to respond to these targets by pressing ‘0’ on the keypad with their right index finger. Participants were not told what the nogo expression would be but were instructed to respond as quickly as possible (while maintaining accuracy) to the target expression and to withhold responding for any other expression (full verbatim instructions for the Emo-GNG task are presented in supplementary material, S3).

**Fig. 2:**
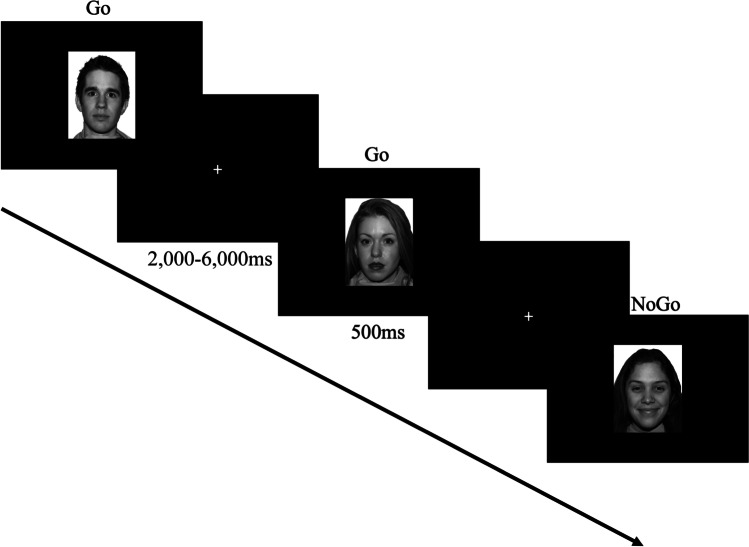
Schematic of the Emo-GNG task events. This example shows three trials in the calm-happy block; participants were instructed to respond as quickly as possible to frequent “go” faces while withholding responses to infrequent “nogo” faces

To induce a prepotent tendency to respond, 73% (35 trials) of the trials in each block were go trials, and 27% (13 trials) were nogo trials. As in previous Emo-GNG tasks (Durston et al., 2002; Hare et al., 2005), trial order was pseudorandom and parametrically balanced to control for the number of go stimuli preceding each nogo stimulus and to ensure that nogo trials occurred equally across the early, middle, and late stages of a block. Stimuli were presented in the centre of the screen for 500 ms at a size of 7.2 cm wide 9.2 cm high. A white fixation cross positioned centrally atop a black background was presented during each interstimulus interval (ISI), which was jittered, ranging from 2,000 to 6,000 ms (M ± SD = 3,708 ms ± 1211 ms). Following the onset of a stimulus there was a 2,000 ms window in which responses were recorded (go trials in which a response was not made within this time window were classed as misses). A schematic of the trial structure is depicted in Fig. 2.

#### Emo-Stroop

Face stimuli comprised the same male and female actors used in the Emo-GNG. The facial expressions used in the Emo-Stroop were angry, happy, and calm (i.e., neutral). Each image was converted to greyscale, with the dimensions of 256 × 329 pixels. Different actors (identities: 7, 14, 20, 38) and emotional expressions (fearful) were used for the practice trials. For each face, the word “angry” or “happy” was superimposed over the bridge of the nose (so as not to obscure any features) in capitalized Arial font at a size of 30 with 10% transparency. The stimuli were presented centrally on the monitor at a size of 13 cm high by 10 cm wide.

The Emo-Stroop took approximately 15 min to complete. This comprised 16 practice trials, followed by three experimental blocks each comprising 48 trials. The blocks were emotion-specific (i.e., angry face, happy face, calm face), and contained an equal number of trials in which the word was “happy” or “angry”. Trial and block orders were randomised. The task was 2-AFC, with participants instructed to respond by pressing the 1 or 2 key on the keyboard with the index and middle finger of their right hand depending upon whether the word was positive (i.e., “happy”) or negative (i.e., “angry”; button mappings were counterbalanced across the sample). Full verbatim instructions for the Emo-Stroop task are presented in supplementary material, S3.

**Fig. 3:**
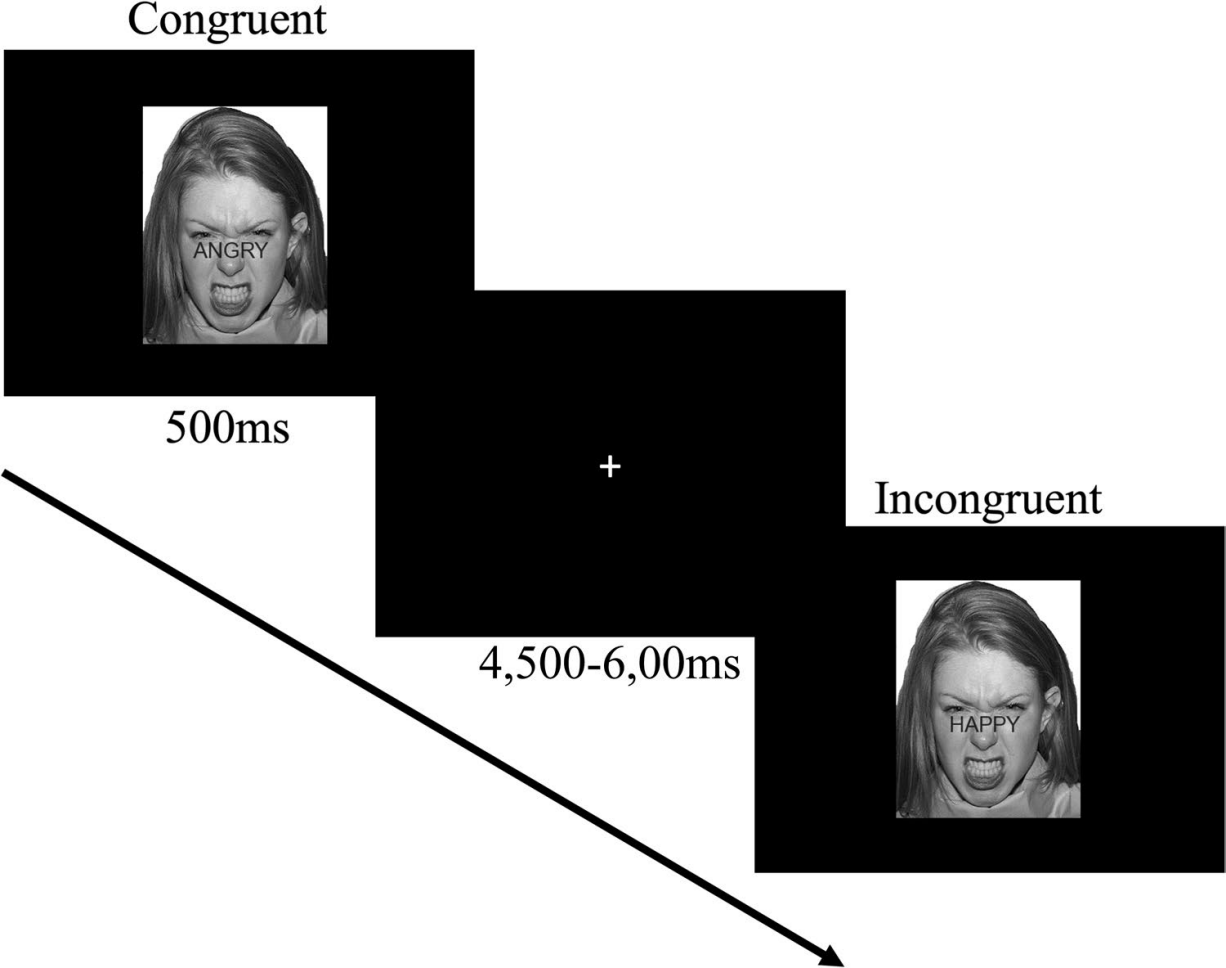
Schematic of events in the Emo-Stroop. This figure depicts an example of a congruent and an incongruent stimulus in the angry face block

Each stimulus was presented for 500 ms, followed by a fixation screen comprising a white cross presented centrally atop a black background. The duration of the fixation screen was jittered, ranging from 4,500 to 6,000 ms (M ± *SD* = 5,250 ms ± 565 ms). Responses were recorded within a 2,500 ms window, which incorporated the 500 ms stimulus presentation and 2,000 ms of the post-stimulus fixation screen. Any trials in which the participant failed to respond within this time window were classed as incorrect. A schematic of the Emo-Stroop task structure is depicted in Fig. 3.

### Data Reduction and Analysis

Bivariate correlations were used to examine the relationship between trait cognitive/affective empathy and the emotion interference effect metrics from the Emo-GNG and Emo-Stroop (see task-specific sections below for details of how these metrics were calculated). Normality of each variable was assessed using Kolmogorov–Smirnov tests. As some variable distributions showed significant deviation from normality, Spearman’s rho is reported for all correlations in order to enable their direct comparability. All correlations are reported as two-tailed, with an alpha level of *p* = 0.05. Data were analysed using SPSS version 27 (IBM Corp., Armonk, N.Y., USA) and Jamovi version 1.6 (https://www.jamovi.org). To ensure that the observed results were not unduly influenced by a small number of outlier cases, the same analyses reported in the “Results” were conducted following the removal of univariate and bivariate outlier cases (see supplementary material, S4). Descriptive statistics for all variables included in the correlation analyses are reported in supplementary material (S5).

#### Emo-GNG

Participants with a mean hit rate (HR) or false alarm rate (FAR) that deviated from the group mean by more than 3*SD were removed as outliers (12 participants). Visual inspection of these data confirmed that these participants had failed to correctly follow task instructions in at least one block (e.g., they always/never responded across all trials and/or evidently confused the go/nogo stimuli). Further, one participant was removed because the necessary questionnaire data was incomplete, leaving a final sample of N = 79, which was subject to analysis.

The key index of task performance on the Emo-GNG was D-prime, which was calculated by subtracting the *z*-transformed FAR from the *z*-transformed HR. As D-prime calculations cannot be performed for values of 1 (i.e., 100% HR/FAR) or 0 (i.e., 0% HR/FAR), any such values were transformed using the formula: 1/(2 N) for values of 0, and 1–1/(2 N) for values of 1. D-prime data were analysed using a repeated-measures ANOVA with trial type (Emo-NoGo/Calm-NoGo) and emotion (happy, sad, disgust) as within-subjects factors. Greenhouse Geisser corrected values are reported where the assumption of sphericity was not satisfied.

The key metric extracted from this task for the correlation analysis was the difference in mean D-prime between the calm-nogo and emo-nogo conditions (calm-nogo D-prime minus emo-nogo D-prime). This metric was termed the “emotion interference effect”. As the different facial expression pairings were balanced across these two conditions, this metric controls for variability in participants’ ability to discriminate between the calm and emotional faces, providing a direct measure of the extent to which their performance was affected by emotional nogo stimuli.

#### Emo-Stroop

An unexpected error in the task program resulted in data for a substantial number of trials being lost for two participants; these cases were removed prior to analysis. Two participants who failed to complete the necessary questionnaire data were also removed. To ensure data quality, participants with an overall mean accuracy more than 3*SD below the group mean were removed (5 cases), leaving a final sample of *N* = 83, which was subject to analysis. Incorrect trials (mean number of trials removed per participant = 12.28, SD = 8.07) and correct trials in which the RT deviated from the group mean by more than 3*SD were removed (mean number of trials removed per participant = 8.08, SD = 10.65). The mean number of trials per block following all removals was 41.21 (SD = 4.76).

The dependent variable in the Emo-Stroop was RT for correct trials. These data were analysed using a repeated-measures ANOVA, with face (angry, happy, calm) and word (angry, happy) as within-subjects factors. Greenhouse Geisser corrected values are reported where the assumption of sphericity was not satisfied. Given the inclusion of a neutral (calm face) control condition, this paradigm enabled the assessment of interference (resulting from incongruent task-irrelevant emotional faces) independently of facilitation (resulting from congruent task-irrelevant emotional faces) effects. The emotion interference effect metric was calculated by subtracting the mean RT for the neutral control condition from the mean RT for the incongruent condition.

## Results

### Emo-GNG

**Fig. 4:**
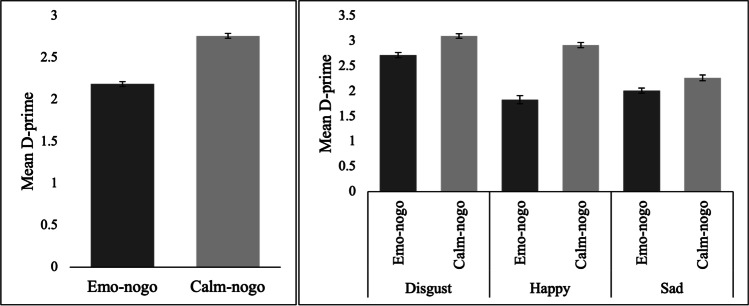
Mean D-prime for Emo-NoGo and Calm-NoGo conditions on the left. Mean D-prime for Emo-NoGo and Calm-NoGo trials for each emotion on the right (see supplementary material S6 for accompanying results). Error bars depict ± 1 within-subjects SEM

A repeated-measures ANOVA demonstrated a significant main effect of trial type, *F*(1, 78) = 96.60, *p* < 0.001, *η*_*p*_^*2*^ = 0.55, and emotion, *F*(1.83, 142.88) = 70.91, *p* < 0.001, *η*_*p*_^*2*^ = 0.48. A significant trial type by emotion interaction was also observed, *F*(2, 156) = 32.52, *p* < 0.001, *η*_*p*_^*2*^ = 0.29. D-prime was significantly higher for calm (*M* ± *SD* = 2.76 ± 0.51) relative to emotional (*M* ± *SD* = 2.19 ± 0.61) nogo trials (*p* < 0.001; Fig. 4, left panel). The post hoc pairwise comparisons for the main effect of emotion and the trial type by emotion interaction are presented in supplementary material (S6).

**Fig. 5:**
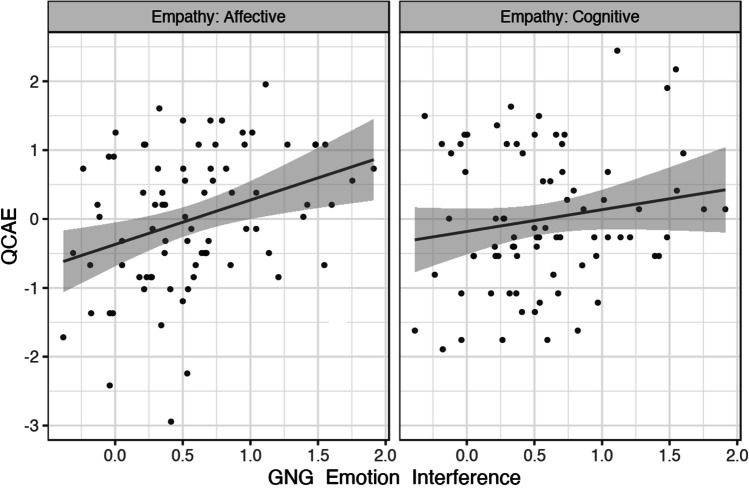
Scatterplot showing the relationship between *z*-transformed cognitive/affective empathy and the Emo-GNG emotion interference effect (calm-nogo D-prime minus emo-nogo D-prime). Affective empathy (left panel) showed a significant positive correlation with the emotion interference effect; cognitive empathy (right panel) was not significantly related to the emotion interference effect

Trait affective empathy was positively correlated with the GNG emotion interference effect, *rho*(77) = 0.34 (CI_95%_[0.12, 0.52]), *p* = 0.003. Cognitive empathy was not significantly related to the GNG emotion interference effect, *rho*(77) = 0.14 (CI_95%_[-0.08, 0.35]), *p* = 0.22. The difference between these correlations was not significant, Steiger’s *Z* =  − 1.63, *p* = 0.10 (two-tailed; Fig. 5).

Given that affective empathy has been found to be associated with improved emotion discrimination under conditions of brief stimulus exposure (e.g., Kang et al., 2017), it is possible that the correlation between affective empathy and the emotion interference effect could have been driven by a higher D-prime in the calm-nogo condition (i.e., improved HR when responding to emotional go targets). A significant negative correlation between affective empathy and D-prime in the emo-nogo condition was observed, *rho*(77) =  − 0.24 (CI_95%_[− 0.44, − 0.02]), *p* = 0.04; affective empathy showed no relationship with D-prime in the calm-nogo condition, *rho*(77) = 0.05 (CI_95%_[− 0.17, 0.27]), *p* = 0.64. These results suggest that the correlation between affective empathy and the emotion interference effect was driven by increased emotional interference in the emo-nogo condition, rather than improved performance in the calm-nogo condition. Cognitive empathy was not significantly related to D-prime in either the emo-nogo, *rho*(77) =  − 0.13 (CI_95%_[− 0.30, 0.10]), *p* = 0.26, or the calm-nogo condition, *rho*(77) =  − 0.02 (CI_95%_[− 0.23, 0.21]), *p* = 0.90.

### Emo-Stroop

**Fig. 6:**
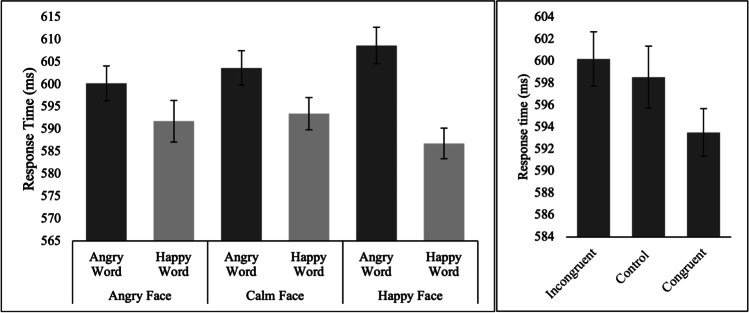
Mean RT across each condition in the Emo-Stroop task (left panel). Mean RT for the incongruent, congruent, and control conditions (right panel). Error bars depict ± 1 within-subjects SEM

Mean accuracy was 91.47% (*SD* = 5.6), which confirms that participants were able to complete the task as instructed. A repeated-measures ANOVA examining the effect of face (angry, happy, calm) and word (angry, happy) on the dependent variable RT demonstrated a significant main effect of word, *F*(1, 82) = 19.05, *p* < 0.001, *η*_*p*_^*2*^ = 0.19, with shorter RTs for “happy” compared to “angry”, but no main effect of face, *F*(2, 164) = 0.13, *p* = 0.88, *η*_*p*_^*2*^ = 0.002. The hypothesised face by word interaction was at trend-level, *F*(2, 164) = 2.31, *p* = 0.10, *η*_*p*_^*2*^ = 0.03. In the calm face condition, RT for the word happy (*M* ± *SD* = 593.44 ms ± 101.22 ms) was significantly shorter than for the word angry (*M* ± *SD* = 603.64 ms ± 100.28 ms; *p* = 0.04). Similarly, in the happy face condition, RT for the word happy (*M* ± *SD* = 586.80 ms ± 102.06 ms) was significantly shorter than for the word angry (*M* ± *SD* = 608.65 ± 101.07 ms; *p* < 0.001). However, in the angry face condition, the difference between RT for the word happy (*M* ± *SD* = 591.75 ms ± 98.13 ms) and the word angry (*M* ± *SD* = 600.19 ms ± 98.64 ms) did not reach significance (*p* = 0.11; Fig. 6, left panel).

In sum, some evidence of emotion interference effects was observed, which reduced the positive word RT advantage in the angry face condition. To test more directly for interference effects, paired-samples *t*-tests were conducted to compare RT across the congruent, incongruent, and control conditions. No significant differences were observed between the control and incongruent conditions (*t*(82) = 0.34, *p* = 0.73). A trend-level difference was observed between the congruent and incongruent conditions, *t*(82) = 1.82, *p* = 0.07 (two-tailed; Fig. 6, right panel).

As per our hypothesis, we examined how trait empathy was associated with individual differences in the magnitude of the emotion interference effect. It is worth noting that the lack of a significant effect at the group level does not necessarily refute the potential presence of significant individual differences (see Hedge et al., 2018 for a relevant discussion). Indeed, prior studies have demonstrated that Emo-Stroop interference and incongruency effects may often be observable only in certain individuals/groups, such as those with high trait/clinical anxiety (e.g., Kalanthroff et al., 2016; Richards et al., 1992).

**Fig. 7:**
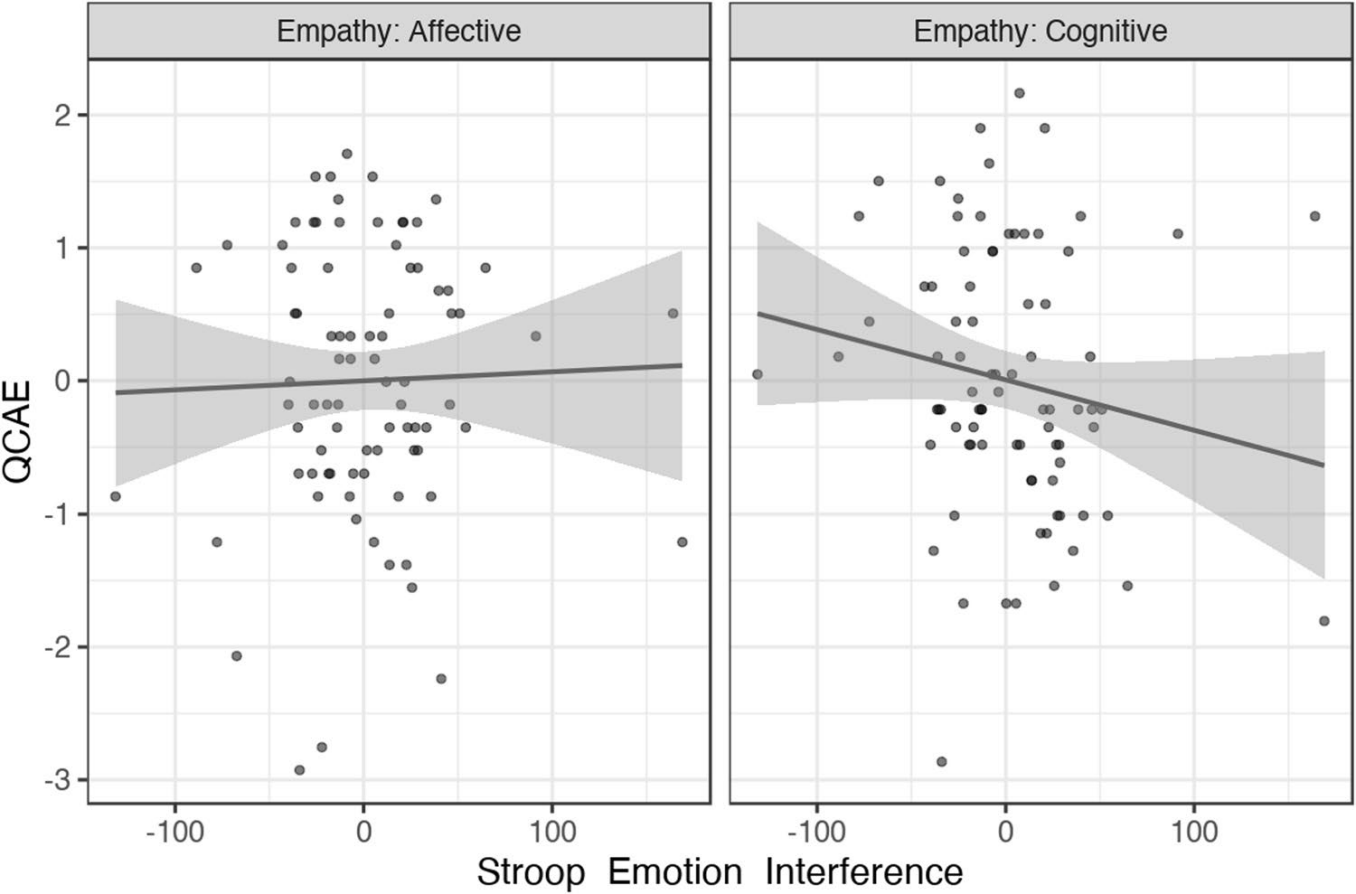
Scatterplot showing the relationship between *z*-transformed cognitive/affective empathy and the Emo-Stroop emotion interference effect (incongruent RT minus control RT). The emotion interference effect was not related to affective empathy (left panel) but showed a significant negative relationship with cognitive empathy (right panel)

Trait cognitive empathy was negatively correlated with the Emo-Stroop emotion interference effect, *rho*(81) =  − 0.24 (CI_95%_[− 0.43, − 0.03]), *p* = 0.03. In contrast, affective empathy showed no relationship with this emotion interference effect, *rho*(81) = 0.003 (CI_95%_[− 0.21, 0.22]), *p* = 0.98. The difference between these correlations was approaching the threshold for significance, Steiger’s *Z* =  − 1.95, *p* = 0.05 (two-tailed; Fig. 7).

## Discussion

Across two studies, the relationship between trait empathy and emotion regulation was examined. It was predicted that greater cognitive empathy would be associated with improved emotion regulation (i.e., reduced emotion dysregulation), whereas greater affective empathy would be associated with increased difficulties in emotion regulation (i.e., increased emotion dysregulation). These predictions were largely borne out by the data, with a few caveats as discussed below.

In study 1, trait cognitive and affective empathy showed divergent patterns in their relationships with self-reported emotion dysregulation. Cognitive empathy was negatively related to overall levels of emotion dysregulation, suggesting that those with greater cognitive empathy experience fewer difficulties with emotion regulation. In contrast, affective empathy did not show a significant relationship with overall levels of emotion dysregulation. With the exception of the Clarity subscale, trait cognitive empathy and affective empathy shared significantly different relationships with each subscale of the DERS. These findings are broadly consistent with those reported by Contardi et al. (2016), who observed similar relationships between trait empathy and emotion dysregulation in a sample of Italian students. Our findings provide support for this work by replicating the findings in a UK sample; furthermore, the exploratory subscale analysis provides greater specificity as to the relationship that cognitive and affective empathy share with different aspects of emotion dysregulation.

Building upon the findings of study 1, the second study examined how the same trait empathy measures were associated with performance-based metrics of emotion regulation. We used the magnitude of emotion interference effects (i.e., the extent to which inhibitory control was affected by emotional relative to neutral stimuli) in an Emo-GNG and an Emo-Stroop task as a proxy measure of participants' implicit emotion regulation abilities. Affective empathy was positively correlated with the emotion interference effect in the Emo-GNG, which suggests that higher affective empathy was associated with greater difficulty regulating impulsive behaviours in the presence of emotional distractors. In contrast, no relationship was observed between trait cognitive empathy and the Emo-GNG emotion interference effect. In the Emo-Stroop, higher cognitive empathy was associated with reduced emotional interference, which suggests that individuals with higher cognitive empathy were more efficient in regulating the potential interference caused by task-irrelevant emotional faces. No relationship was observed between affective empathy and the Emo-Stroop interference effect.

On the basis of prior literature, we had predicted that the emotion interference effects from both tasks would show a negative relationship with cognitive empathy, and a positive relationship with affective empathy. However, this hypothesis was only partially supported, since cognitive and affective empathy were associated with different emotion interference effects. We speculate that the Emo-GNG and Emo-Stroop tasks used in study 2 may be assessing different processes relevant to emotion regulation, which could have differential relevance to cognitive and affective empathy, respectively. It has been asserted that the GNG task assesses response inhibition whereas the Stroop assesses conflict resolution, which reflect related but dissociable aspects of cognitive control (Nee et al., 2007; Swick et al., 2011; see also supplementary material S7, where we report no inter-relationship between these task metrics). The differential results for these tasks could reflect the extent to which they each provide a ‘pure’ measure of a unitary component of cognitive control. While the GNG provides a relatively pure measure of inhibition, the Stroop may also assess other aspects of cognitive control not captured by the GNG, such as shifting and updating (Miyake et al., 2000).

Additionally, these divergent findings may be driven by differences in the task-relevance of the emotional face stimuli in these two tasks. Given that the faces in the Emo-Stroop were always task-irrelevant, it could be that participants were able to focus their attention more fixedly on the target words, reducing the likelihood for trait affective empathy to modulate task effects. As participants were required to actively attend to the faces in order to perform successfully on the Emo-GNG task, such an approach would not have been possible. A recent study found that affective empathy was associated with greater self-reported ‘attentional focusing’ abilities but poorer ‘attentional shifting’ abilities (Goodhew & Edwards, 2020), which is consistent with the above interpretation.

Despite the caveats mentioned above, study 2 task results provide evidence to suggest that higher trait cognitive empathy is associated with improved implicit emotion regulation abilities, whereas higher trait affective empathy may be associated with a diminished capacity to regulate emotional influences on cognitive control processes. Given the differences in the way in which emotion regulation was assessed across studies 1 and 2, we are cautious in making strong generalizability inferences from their results. That being said, the findings of study 2 are broadly consistent with those of study 1, where higher cognitive empathy was associated with reduced emotion dysregulation. While affective empathy was not significantly related to overall emotion dysregulation in study 1, it did show positive relationships with subscales assessing difficulties in certain aspects of emotion regulation, such as maintaining focus on goal-directed behaviours.

While the relationships between the two dimensions of empathy and facets of self-reported emotion dysregulation were broadly in opposing directions, cognitive and affective empathy were both negatively associated with difficulties with emotional awareness. This observation is consistent with the interpretation that individuals with greater empathy show a heightened awareness and understanding of their own emotional experiences (Frith & Frith, 2003; Happé & Frith, 1996; Hobson, 2010; Rieffe & Camodeca, 2016), which is a key competency supporting adaptive emotion regulation (Gratz & Roemer, 2004; Gross, 2015).

Prior theoretical and empirical work suggests that the cognitive component of empathy is reliant upon various cognitive control processes, which support the ability to take another’s perspective and make accurate inferences about their mental/emotional state (Carlson et al., 2004; Decety & Sommerville, 2003; Hansen, 2011; Mutter et al., 2006). Given that many forms of adaptive emotion regulation are reliant upon similar processes and neural architecture to those that support cognitive empathy (Buhle et al., 2014; Hendricks & Buchanan, 2016; McRae et al., 2012; Schmeichel & Demaree, 2010), the results of the current studies could reflect the fact that higher cognitive empathy is associated with improved efficiency of the cognitive control processes that also underlie the ability to regulate one’s emotions.

Rather than making any causal attributions to the observed association between cognitive empathy and emotion regulation, we believe that the relationship between these constructs is likely bi-directional in nature. Greater cognitive empathy abilities may support emotion regulation and vice versa. For instance, in an emotional situation, an individual with heightened emotion regulation abilities may have greater cognitive resources available with which to make accurate inferences about others’ states. Similarly, an individual who is more adept in the cognitively demanding task of taking others’ perspectives, may be better able to adopt a more distanced self-perspective in emotional situations, which could facilitate the use of more adaptive regulation strategies such as reappraisal (Kross & Ayduk, 2011; Ochsner & Gross, 2008; Wallace-Hadrill & Kamboj, 2016).

In contrast to cognitive empathy, affective empathy was positively associated with emotion interference effects in the Emo-GNG task and showed positive relationships with DERS subscales that assessed difficulties in managing emotions and maintaining focus on goal-directed behaviours. These findings could reflect a heightened propensity for spontaneous facial mimicry (SFM) and increased emotional reactivity in individuals with higher affective empathy (Eisenberg et al., 1989; Davis, 1983; Rueckert et al., 2011; Sonnby-Borgstrom, 2002; Sato et al., 2013; Wild et al., 2001), which results in increased interference on cognitive control processes necessary for certain aspects of emotion regulation (Tottenham et al., 2011).

The present findings represent an important step towards further elucidating the relationship between the various component processes associated with empathy and emotion regulation. However, it is worth noting the limitations of the current work that future studies could address. First, while emotion regulation was assessed using a combination of self-report trait measures and more objective performance-based metrics, in both studies, empathy was assessed using a self-report questionnaire. While helpful in understanding respondents’ self-perceptions of their own abilities (Dziobek et al., 2008), given that many of the processes associated with empathy are thought to occur on an implicit level (e.g., Decety & Jackson, 2004; Pfeifer et al., 2008; Singer & Lamm, 2009), one could argue that certain features of this construct may be difficult to assess accurately via introspection (Kagan, 1988). Further, self-report empathy measures rely upon retrospective self-reporting, which may leave them susceptible to inaccuracies and/or response biases (Moskowitz, 1986) such as socially desirable responding (Gerdes et al., 2010; Paulhus, 1991). A further limitation of using only trait measures is that they may not accurately capture ability-based components of empathy, which may be more amenable to measurement using performance-based task approaches. Indeed, prior work highlights a lack of convergence between trait and task measures of empathy (e.g., Melchers et al., 2015), which could suggest that these approaches are assessing slightly distinct latent constructs.

While the difference metrics we derived from the tasks in study 2 were designed to minimise the impact of individual differences in general cognitive control, it would be useful for future work to examine the relationship that cognitive and affective empathy share with emotional as well as non-emotional versions of similar cognitive control tasks. Additionally, given the evidence of positive relationships between intelligence and cognitive control abilities (e.g., Checa & Fernandez-Berrocal, 2015; Shamosh & Gray, 2008), controlling for IQ in future work may also help to shed more light on the potential mechanisms that underlie the relationships observed in these studies.

An additional caveat comes from the lack of an overall emotion interference effect in the Emo-Stroop task. While it does not preclude us from drawing inferences about individual differences, it points to the possibility that the condition-related variability in this version of the task may be significantly smaller than the between-subject variability (Hedge et al., 2018). The decision to use the affective faces, rather than the affective words, as the distractor stimuli was based on prior work which demonstrated greater interference effects for face relative to word distractors (Beall & Herbert, 2008). However, other studies have observed contrary results (Ovaysikia et al., 2011), and future work might seek to examine the relative differences in condition-related effects and between-subject variability in these effects across different Emo-Stroop task variants.

Finally, consideration should be given to our characterization of these tasks as implicit regulation. The explicit goal for these tasks was to respond as quickly and accurately as possible. To achieve this explicit goal, participants would have had to regulate the potential interference caused by the emotional distractors. According to Braunstein et al.’s (2017) framework, such ‘incidental’ regulation would be considered implicit. Accordingly, we characterize both of these tasks as assessing implicit emotion regulation. The distinction between implicit and explicit processes is by no means a binary one (Gyurak et al., 2011; Koole et al., 2015), and it would be useful for future work to examine how cognitive and affective empathy relate to regulatory processes that lie at different points along the continuum between implicit and explicit. Similarly, future work should take into account whether different stimuli used as emotional distractors (e.g., IAPS images, threat of shock) yield similar associations with empathy.

In conclusion, the current studies provide new evidence on the relationship that the cognitive and affective dimensions of empathy share with emotion regulation. While greater cognitive empathy was broadly associated with improved emotion regulation abilities based on both self-report and task-based measures, greater affective empathy was associated with increased difficulties with specific aspects of emotion regulation. Given the myriad subprocesses with which both of these constructs are related, coupled with the diverse range of tools used in their assessment, future work should seek to test the generalizability of these findings by examining these relationships using different trait and/or task measures of empathy and emotion regulation to those used in the current studies. An additional avenue for future research would be to explore these relationships in populations known to exhibit atypicalities in empathy and/or emotion regulation, such as individuals with autism spectrum conditions or psychopathy.

## Supplementary Information

Below is the link to the electronic supplementary material.

Supplementary file1 (DOCX 23 KB)

## Additional Information

### Data Availability

The datasets analysed during the current studies are available in the University of Reading Data Archive, 10.17864/1947.305.
