# Supplementary material for: Cognitive and Affective Empathy Relate Differentially to Emotion Regulation
Source: Affect Sci. 2021 Nov 15;3(1):118–34. doi: 10.1007/s42761-021-00062-w (PMC8989800; doi:10.1007/s42761-021-00062-w)
Supplement: Supplementary file 1 — Supplementary file1 (DOCX 23 KB) [file 42761_2021_62_MOESM1_ESM.docx]

**Cognitive and affective empathy relate differentially to emotion regulation**

Nicholas M. Thompson, Carien M. van Reekum, Bhismadev Chakrabarti

**Supplementary Material**

**S1. Study 1 correlation results following outlier removal**

Reported here are the main correlation results from study 1 following the removal of outlier cases. Normality was assessed using Kolmogorov Smirnov tests; Spearman’s rho coefficients are reported as some variable distributions showed significant deviation from normality. Univariate and bivariate outliers were identified using a criterion of 3*IQR and Cook’s distance > 4/N (Bollen & Jackson, 1990), respectively (number of outliers removed <= 6).

|  | DERS-Total |
| --- | --- |
| Cognitive empathy (QCAE) | -.22 (*p* = .01) |
| Affective empathy (QCAE) | .09 (*p* = .32) |

To examine the difference between these correlations in a manner comparable to the Z tests reported in the main manuscript, any cases identified as a bivariate outlier in either relationship were removed for both correlations. The difference between correlations was tested on this maximum dependent (i.e. fully overlapping) sample using a Steiger’s Z test. These two correlations were significantly different, Z = -2.95, *p* = .002.

**S2. Study 1 descriptive statistics for the DERS subscales**

|  | Mean (SD) | Skewness^a^ | Kurtosis^b^ |
| --- | --- | --- | --- |
| DERS-Awareness | 6.63 (2.41) | 0.43 | -0.37 |
| DERS-Clarity | 6.25 (2.76) | 1.00 | 0.37 |
| DERS-Non-acceptance | 7.33 (3.24) | 0.57 | -0.72 |
| DERS-Goals | 10.2 (3.22) | -0.12 | -1.01 |
| DERS-Impulse | 5.92 (3.01) | 1.14 | 0.72 |
| DERS-Strategies | 6.72 (3.08) | 0.86 | -0.11 |

^a^Skewness Standard Error = 0.21

^b^Kurtosis Standard Error = 0.41

**S3. Participant instructions for the Emo-GNG and Emo-Stroop task**

**Emo-GNG Task Instructions**

On each trial a face displaying an emotional expression will be presented on screen. You must press the ‘0’ key with the index finger of your right hand as fast as you can whenever you see a face displaying a particular emotional expression. Do NOT press the button when you see any other expression. At the start of each block you will be told which expression you should respond to.

**Example of Emo-GNG block instructions:**

Press the ‘0’ key as fast as you can whenever you see a HAPPY face. Do NOT

press for any other faces; only the HAPPY faces.”

**Emo-Stroop Task Instructions**

On each trial a WORD and a FACE will be presented on screen. You must press a button on the keyboard to state whether the WORD is positively or negatively valenced. Try to ignore the face and respond as quickly as possible to the word.

Press ‘1’ if the WORD is POSITIVE

Press ‘2’ if the WORD is NEGATIVE

Please press the appropriate key using either the index or middle finger of your right hand. Remember to respond to the word as fast as you can without making mistakes.

**S4. Study 2 correlation results following outlier removal**

Reported here are the main correlation results from study 2 examining the relationship between trait empathy and task measures of implicit emotion regulation (Emo-GNG & Emo-Stroop), with outlier cases removed. Normality was assessed using Kolmogorov Smirnov tests; Spearman’s rho coefficients are reported as some variable distributions showed significant deviation from normality. Univariate and bivariate outliers were identified using the same criteria as reported in S1 (number of outlier cases removed <= 8). Due to some

|  | Emo-GNG emotion interference effect | Emo-Stroop emotion interference effect |
| --- | --- | --- |
| Cognitive empathy (QCAE) | .08 (*p* = .49) | -.25 (*p* = .03) |
| Affective empathy (QCAE) | .33 (*p* = .003) | -.02 (*p* = .84) |

To examine the difference between these correlations in a manner comparable to the Z tests reported in the main manuscript, the same process for removing outlier cases described in S1 was used here. For the Emo-GNG task, the correlations for cognitive and affective empathy were significantly different, Z = 1.81, *p* = .04. For the Emo-Stroop task, the correlations for cognitive and affective empathy were not significantly different, Z = -1.09, *p* = .14.

**S5. Study 2 descriptive statistics for all correlation variables**

|  | Mean (SD) | Skewness^a^ | Kurtosis^b^ |
| --- | --- | --- | --- |
| QCAE - Cognitive | 57.96 (7.38) | 0.21 | -0.51 |
| QCAE - Affective | 35.82 (5.72) | -0.47 | 0.01 |
| GNG-Emotion interference | 0.57 (0.52) | 0.51 | -0.18 |

^a^Skewness Standard Error = 0.27

^b^Kurtosis Standard Error = 0.54

|  | Mean (SD) | Skewness^a^ | Kurtosis^b^ |
| --- | --- | --- | --- |
| QCAE - Cognitive | 57.64 (7.56) | -0.08 | -0.21 |
| QCAE - Affective | 35.05 (5.83) | -0.56 | 0.24 |
| Stroop-Emotion interference | 1.66 (44.1) | 0.84 | 4.18 |

^a^Skewness Standard Error = 0.26

^b^Kurtosis Standard Error = 0.52

**S6. Study 2 Emo-GNG emotion main effect & target*emotion interaction post-hoc tests**

Post-hoc pairwise comparisons with Bonferroni adjustment revealed that D-prime for disgust (*M ± SD* = 2.90 *±* 0.58) was significantly higher than for happy (*M ± SD* = 2.37 *±* 0.63) and sad (*M ± SD* = 2.14 *±* 0.59) (both *p* < .001). D-prime for happy was significantly higher than for sad (*p* = .004). Regardless of the emotion, D-prime was always higher for blocks where the nogo stimulus was a calm face: Disgust (calm-nogo *M ± SD* = 3.09 *±* 0.57; emo-nogo *M ± SD* = 2.72 *±* 0.72, *p* < .001), happy (calm-nogo *M ± SD* = 2.92 *±*  0.61; emo-nogo *M ± SD* = 1.83 *±* 0.94, *p* < .001), sad (calm-nogo *M ± SD* = 2.27 *±* 0.72; emo-nogo *M ± SD* = 2.01 *±* 0.67 *p* = .003). For calm nogo trials, disgust was significantly higher than happy (*p* = .03) and sad (*p* < .001), and happy D-prime was significantly higher than sad (*p* < .001). For emo nogo trials, D-prime for disgust was higher than for happy and sad (both *p* < .001), but there was no difference between happy and sad (*p* = .27).

**S7. Emo-GNG and Emo-Stroop task metrics correlation**

Reported below are the full sample correlations between the emotion interference metrics from the Emo-GNG and Emo-Stroop tasks.

|  | Emo-Stroop emotion interference effect |
| --- | --- |
| Emo-GNG emotion interference effect | .02 (*p* = .88) |
